# Implementing complaint-directed mini-interventions for depressive complaints in primary care to increase participation among patients with a lower socioeconomic status: design of a cluster randomised controlled trial

**DOI:** 10.1186/s13063-019-3890-6

**Published:** 2020-01-10

**Authors:** Stephanie S. Leone, Suzanne Lokman, Brigitte Boon, Agnes van der Poel, Filip Smit, Moniek Zijlstra-Vlasveld, Odile Smeets

**Affiliations:** 1 Department of Public Mental Health, Trimbos Institute: Netherlands Institute of Mental Health and Addiction, Utrecht, The Netherlands; 2 Tranzo, Tilburg School of Social and Behavioral Sciences, Tilburg University, Tilburg, The Netherlands; 3 Academy Het Dorp, Arnhem, The Netherlands; 4 Siza, Arnhem, The Netherlands; 5 Department of Epidemiology and Biostatistics and Department of Clinical, Neuro and Developmental Psychology, Public Health research institute, University Medical Centers Amsterdam, Amsterdam, Netherlands; 6 ZonMw, the Netherlands Organisation for Health Research and Development, The Hague, The Netherlands

**Keywords:** E-health, Implementation, Mental health, Depression, Primary care, General practice, Lower socioeconomic status

## Abstract

**Background:**

Depression is a major public health concern. E-health interventions for preventing and reducing depressive complaints have proven to be effective, and have the potential to make (mental) health care more accessible and efficient. However, the reach of these interventions needs to be improved, especially among people with a lower socioeconomic status (SES). Stimulating and supporting implementation of e-health in primary care, and offering guidance from general practice nurses (GP nurses) may be important strategies to achieve this.

**Methods/design:**

The online ‘Complaint Directed Mini-Interventions’ (CDMIs) for stress, sleep and worry complaints, which were found to be (cost-)effective in a self-guided format, will be implemented in the primary care setting using a blended care format (i.e. combining e-health with face-to-face sessions) with minimal guidance provided by the GP nurse. The main aim is to evaluate whether a SES-sensitive implementation strategy improves the participation rate (i.e. reach) of lower-SES patients in the blended online CDMIs as compared to a regular implementation strategy in a cluster randomised controlled trial. Randomisation will occur at the level of the GP nurse, and 228 patients will be included in the study. The primary outcome is the participation rate (completing at least one face-to-face session and two online exercises) of the lower-SES target group. It is hypothesised that this percentage will be higher in the SES-sensitive group as compared to the regular group. Secondary objectives are to evaluate the implementation process, to monitor and evaluate psychological complaints (depression, sleep, stress, worry and anxiety) and well-being over time. Patient assessments will take place at baseline, 3 and 12 months post baseline.

**Discussion:**

This study should contribute to our knowledge of reaching the lower-SES groups with a brief and complaint-specific blended approach for depressive complaints in primary care. It should also further our knowledge on successful strategies to implement depression prevention in primary care.

**Trial registration:**

Netherlands Trial Register, ID: NL6595. Registered on 12 November 2017.

## Background

Depression is a major public health concern associated with individual suffering, impaired functioning and substantial societal costs [1–3]. There is growing evidence that interventions targeting (symptoms of) depression are effective, and that preventing and reducing depressive symptoms could be a viable option [4]. However, the reach of these interventions is only modest, especially among people with a lower socioeconomic status (SES) [5–7]. This is of importance as people with a lower SES seem to be particularly at risk for depression. In the Netherlands, for example, the life-time prevalence of depression is higher among those with a low income (21.4%) as compared to those with a high income (15.2%) [8].

However, e-health interventions (interventions offered over the Internet) for depression and depressive symptoms may reach large populations. These interventions are more accessible and more flexible in use than regular face-to-face interventions. Nonetheless, participants in online interventions are primarily highly educated [9], while those with lower education levels are reached to a lesser extent. This implies that those with a medium or low educational level are not reached as widely with these interventions. If uptake is achieved, evidence shows that adherence to online interventions needs improvement [9–11], especially if these interventions are to make a greater impact on population health. Although self-guided online interventions can be effective, providing guidance to patients may improve not only adherence, but also the effectiveness of such interventions [12–14]. Embedding (preventive) online interventions for depressive complaints in routine care combined with guidance from a health care professional may also provide an important strategy for implementing available online interventions among lower-SES populations.

In Dutch primary care, it is the task of the general practitioner (GPs) to provide mental health care in case of mild to moderate mental health complaints. The GP mental health nurse (GP nurse), who works alongside the GP, plays a pivotal role in delivering mental health care in the GP setting. Because the GP nurse focusses on mental health and provides longer consultations than GPs, the GP nurse is well-placed to offer guidance in e-health interventions for depression. Moreover, GP nurses report a favourable attitude towards e-health and may already offer some type of e-health intervention for mental health [15, 16]. Nonetheless, GP nurses indicate that there is room for improvement. One frequently mentioned problem is the lack of suitable e-health interventions, specifically for low-educated patients. In addition, GP nurses prefer interventions that can be tailored to the patient’s personal complaints which is often not possible in current standardised (one-size-fits-all) interventions. Early dropout of patients and too few possibilities to familiarise themselves with e-health interventions were other frequently mentioned problems [16]. In sum, e-health interventions need to be integrated in the GP setting and need to be matched better to the personal needs of patients, in order to reach more patients, especially those with a lower SES.

In the present study, the online ‘Complaint Directed Mini-Interventions’ (CDMIs), which were found to be (cost-)effective in a self-guided format [17, 18], will be implemented in the primary care setting using a blended care format (i.e. combining the e-health intervention with supporting face-to-face sessions that will be provided by the GP nurse). The online CDMIs are short, low-threshold unguided web-based interventions that target depressive complaints by focussing on highly prevalent complaints that are demonstrably associated with depression and have a substantial economic impact: stress, sleep problems and worry [19]. There are three different CDMIs: ‘Sleep better’, ‘Stress less’ and ‘Worry less’, and each CDMI contains various modules and exercises (see [18] for more information). A feature of the CDMI intervention framework is that patients can choose the complaints that they want to focus on (by choosing the CDMIs that they want to use), and it also allows patients to choose the modules and exercises that they want to do and in what order (pick and mix). The CDMIs are based on cognitive-behavioural techniques as well as elements from solution-focussed therapy, mindfulness and positive psychology. They were developed to be as easy to understand and use as possible. They are focussed on ‘doing’ rather than ‘reading’ with the aim to appeal to a broad range of people, including those with a lower educational level. However, in line with other studies of e-health interventions for depression [9, 20], mainly individuals with a high education level or income participated in the randomised controlled trial (RCT) of the self-guided format (more than 70%). This indicates that there is a clear need for implementation strategies that increase the reach of e-health interventions for depression, including the CDMIs, particularly among lower-SES groups. They may particularly benefit from a more proactive strategy [21], including more proactive and intensive guidance, and additional strategies that take the specific needs of this target group into consideration such as low(er) health-literacy skills [22, 23]. Therefore, a SES-sensitive implementation strategy will be developed in collaboration with primary care professionals and patients, and will be compared to a regular implementation strategy in a randomised controlled trial.

## Methods/design

This protocol was developed in accordance with the Standard Protocol Items: Recommendations for Interventional Trials (SPIRIT) Statement. See Additional file 1 for the SPIRIT Checklist.

### Aim

The aim of this study is to evaluate whether a SES-sensitive implementation strategy to implement the online CDMIs in primary care improves the percentage of lower-SES participants as compared to a regular implementation strategy. The hypothesis is that the percentage of lower-SES participants in the online CDMIs will be higher in the SES-sensitive implementation strategy group as compared to the regular implementation strategy group.

### Design

**Fig. 1:**
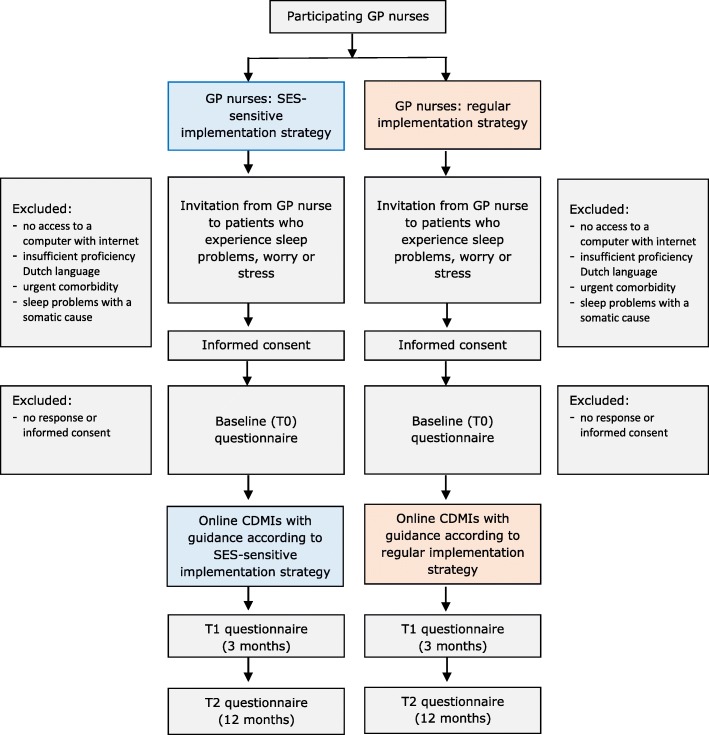
Flowchart of the study

A pragmatic cluster randomised controlled trial with two parallel groups will be conducted in which the two implementation strategies will be compared. As the GP nurse plays a key role in the implementation strategies, randomisation will take place at the level of the GP nurse in order to avoid contamination between the two implementation strategy conditions. Thus, each GP nurse will be randomised to one of the two implementation strategy groups. GP nurses cannot be blinded to the implementation strategy that they are allocated to because they will be aware whether their allocated implementation comprises specific components aimed at reaching lower-SES patients. The randomisation status will not be disclosed to patients. It is worth noting that the online CDMIs will be implemented in both conditions, meaning that the intervention will not be withheld from any patients participating in the study. Moreover, patients are free to receive any other type of care during the study. Participating patients will be assessed at baseline (T0), 3 months after baseline (T1) and 12 months after baseline (T2). Assessments among participating GP nurses will take place at two time points: at baseline, and 6 months after baseline. See Fig. 1 for the flowchart of the study and Table 1 for an overview of the schedule of enrolment, interventions and assessments in accordance with the SPIRIT Statement. This study is registered at the Netherlands Trial Register (NL6595) and was approved by the Medical Ethics Committee of the VU University Medical Center.

**Table 1:**
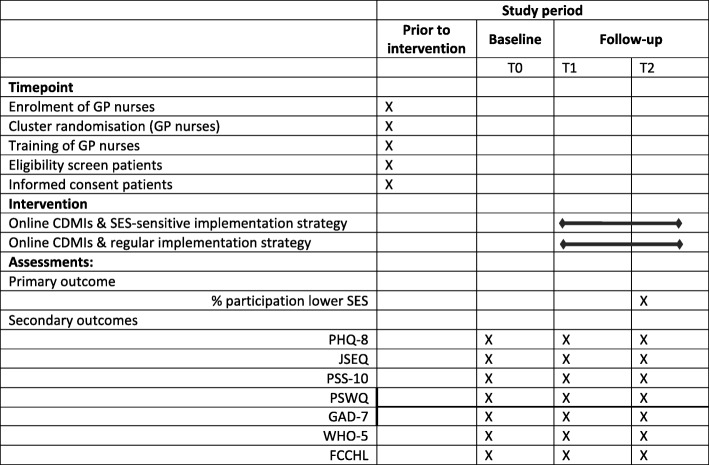
Schedule of enrolment, interventions and assessments

### Randomisation

Randomisation will be carried out by an independent statistician using a computer-generated schedule. Randomness will be assured by the use of true random numbers of Random.org (https://www.random.org). Randomisation will be stratified for the SES (low SES, medium SES, high SES) of the neighborhood in which the GP nurses work in order to balance the distribution of this factor across the two study groups. The SES status level of the neighbourhood (low SES, medium SES, high SES) will be used as the stratification variable. The neighborhood SES level is linked to the four-digit postal codes of the neighbourhood where the GP nurse works and matched to the 2016 social status scores as determined by the Netherlands Institute for Social Research [24].

### Eligibility criteria and recruitment

#### GP nurses

GP nurses from two organisations will participate in this study. One of the organisations is a primary care health service provider in the Almere region of the Netherlands. It elected to participate in this study with the whole organisation (all primary care practices and their GP nurses). The other organisation provides GP nurses serving GP practices in the Rotterdam-Rijnmond area of the Netherlands. This organisation recruited GP nurses willing to participate (via email) after members of the research team gave a short presentation about the study. The GP nurses will recruit patients from the general care practice(s) where they work according to the implementation strategy (i.e. the regular or SES-sensitive implementation strategy) to which they were randomly assigned.

#### Patients

Primary care patients of the GP nurses involved in the study are eligible for participation, when they:

- Are 18 years of age or older
- Experience worry, stress or sleep problems (the latter without a medical cause such as sleep apnea as ascertained by the GP or GP nurse)
- Have access to the Internet
- Have sufficient proficiency of the Dutch language
- Have no acute or urgent comorbidity
- Provide informed consent.

In view of the recently published report about the risk of including suicidal patients in RCT studies [25], we will not exclude patients with suicidal ideation. However, suicidal thoughts will be assessed at baseline, after 3 and after 12 months. To this end, item 15 of the Web Screening Questionnaire (WSQ) will be used [26]. This question is comparable with item 9 of the PHQ-9, but the answers give more detail about the degree of active suicidal ideation, which is not well reflected by item 9 of the PHQ-9 [27]. The item is scored on 4-point scale (0 = definitely not, 3 = I would do it given the opportunity). If participants score 3 on this item they will be advised to discuss the suicidal thoughts with their GP nurse or to contact the anonymous online platform for people with suicidal thoughts or behaviours (www.113Online.nl). The GP nurses can check the answer of this item in the CDMI monitoring system (see below).

#### Inclusion procedure

The GP nurse will inform eligible patients about the online CDMIs and the study during a consultation. If the patient shows interest in participating, the GP nurse will hand over an envelope with: (1) an information brochure about the online CDMIs and (2) an information letter about the study. In addition, the GP nurse will send an invitation to the patient to participate in the online CDMIs by email (sent from the monitoring system). The patients will subsequently receive an email with information about the study and are asked if they are willing to participate in the study. If this is the case, they can click on a button which directs them to the informed consent form. They can then provide consent by clicking on the ‘I consent’ button. Following this, the patients are directed to a webpage where they can create their own online CDMI account by providing their email address, password and gender. After logging in to the online CDMIs for the first time they will be asked to complete the (online) baseline questionnaire. Data collected for research purposes will be stored in a database using a unique respondent number for each individual patient. Email addresses will be stored in a separate database on a separate server. Data will also be used for clinical purposes within the monitoring system. Data processing agreements will be put in place with the relevant parties, besides the research team, who have access to the data for this purpose (ICT parties/monitoring system provider and primary care organisations). Patients who indicate that they do not wish to participate in the study are offered the opportunity (by clicking on a link) to use the self-help version of the online CDMIs (without the GP nurse being able to monitor progress and provide guidance using a monitoring system). The flowchart of the study is presented in Fig. 1.

#### Monitoring system

To facilitate the implementation of the blended CDMI format in primary care, the online CDMIs will be linked to a monitoring system allowing the GP nurses to check on the patient’s progress when using the CDMIs and to track complaints over time. The monitoring system will provide the GP nurse with insight into the intervention use of their patients (e.g. answer to questionnaires, diary entries, number of logins, number of completed exercises), and allows them to send messages to their patients.

#### Implementation strategies

Successful implementation of e-health is not only related to the patient-related factors, but also to the intentions and motivations of health care providers and their management [28, 29]. This means that GP nurses play a crucial role in the implementation process. In this study, the main focus of the implementation strategies is aimed at the GP nurses and the patients. That being said, management-level representatives of the two participating organisations will be actively involved during the implementation process and will also be members of an Advisory Committee that will be installed for the purpose of this project. This will allow for an ongoing dialogue about relevant organisational aspects and possibilities for (organisational) support or even change throughout the implementation process. The main aim of the Advisory Committee is to advise the project team on a range of content- and implementation-related issues in order to facilitate the execution of the project. Their role is thus advisory: their suggestions will be carefully considered, yet they do not have decision-making power. Besides the representatives of the participating organisations, members of the Advisory Committee will be independent of the trial. Given the goal of our project to compare two implementation strategies for the online CDMIs (an intervention that has been studied in a previous trial), and the implementation-related primary outcome of this study, we did not install a data monitoring committee. A DMC was also not part of the approved protocol.

The implementation strategy development process was informed by the implementation model of Grol & Wensing [30]. Before developing and selecting specific implementation strategies, this model suggests: (1) developing a proposal for change (in this study the adapted blended CDMIs), (2) an analysis of actual performance and targets for change and (3) a problem analysis including characteristics of the target group, the setting, and possible barriers and facilitators for implementation. With respect to step 2, as noted above, the uptake of e-health interventions leaves a lot to be desired, especially in lower-SES populations. Therefore, increasing the participation rates of people with a lower educational level is the main target for change in this study (see sections ‘Primary outcome’, and ‘Sample size’ calculation for more details). For the problem analysis, interviews and focus group meetings were conducted with people with a low-SES background, people with (a former) depression, GP nurses and other professionals working with people with mental health problems or a low SES, and this was supplemented with findings from the literature. Results from the problem analysis were also used for adapting the actual CDMIs courses for use in primary care.

Based on the findings of the previous three steps, we developed and selected sub-strategies (step 4) to create the overall regular implementation strategy and the SES-sensitive implementation strategy. As can be discerned from the description of the regular and the SES-sensitive implementation strategies outlined below, the same general (sub-)strategies are applied in both implementation strategies, but some are tailored to be SES-sensitive in the SES-sensitive implementation strategy. The implementation strategies will be evaluated in step 5 using the RCT described in this protocol. The model also suggests a continuous evaluation of the implementation process, and adapting it if necessary.

##### Regular implementation strategy

Prior to the start of the study, the research team will visit the implementation locations and give a presentation during GP nurse team meetings to garner interest in, and support for, the study.

Participating GP nurses who have been randomised to the regular implementation strategy will then be invited to attend a 2-h training in which they will:

- ▪ Learn about the online CDMIs, the related monitoring system and guidelines for the recruitment and guidance process
- ▪ Practice using the online CDMIs and its monitoring system
- ▪ Discuss possible barriers and facilitators for implementation

To be able to tailor the guidance to specific individual needs of patients, GP nurses in the control condition will be free in the amount of guidance they give. The only requirement is that they provide at least one face-to-face consultation. However, the basic advice suggested in the implementation manual is to schedule 3 consultations: at the start, midway and at the end of the period in which the CDMIs are used by the patient. It is also up to the GP nurses whether or not to recommend certain modules or exercises within the CDMIs. During the training general guidelines for topics that can be discussed during the consultations (e.g. what kind of instruction to give about the online CDMIs, discussing the diary, giving compliments) as well as indicating potential (motivational) barriers of patients to initiate and maintain usage of the online CDMIs. The GP nurses will be provided with a manual with information on all the topics covered during the training. After a practice period of approximately 2 months, the GP nurses will be invited to participate in a follow-up session, led by the research team, to exchange experiences and discuss potential problems that arose during the practice period.

The research team will provide ongoing support to the GP nurses throughout the implementation period in several ways:

- ▪ A helpdesk service (email or telephone contact) will be provided to support GP nurses to handle technical and implementation-related issues
- ▪ At least two implementation team meetings (led by the research team) will be organised in which the participating GP nurses will be able to exchange their experiences, tips, ideas and to discuss encountered implementation barriers with each other. These meetings will also provide the research team with the opportunity to provide additional information, make adjustments or reiterate important issues if necessary
- ▪ Regular communication from the research team with the GP nurses through newsletters, telephone calls and emails to keep the online CDMIs on the agenda of the GP nurses, and provide ongoing information and tips

The main elements of the regular implementation strategy aimed at the patients are: (1) a brochure with information about the online CDMIs, information about privacy, the study, and required time investment, (2) providing reminders to use the online CDMIs after a lack of activity and (3) receiving support from the GP nurse according to the guidelines for the GP nurse as outlined the CDMI implementation manual.

More information about the regular implementation strategy is provided in Table 2 below.

**Table 2:** Detailed overview of the regular implementation strategy

Adaptation of the online Complaint-Directed Mini-Interventions (CDMIs) to allow use by general practice (GP) nurses in a blended format	
• Developing a monitoring system that allows GP nurses to: ◦ Invite patients to create an account ◦ Monitor use of CDMIs ◦ Track mood and complaints ◦ Provide the opportunity to message patients	
Create awareness of CDMIs and implementation project among GPs and GP nurses	
• Providing an information brochure for GPs to facilitate patient flow to the GP nurse and online CDMIs • Short outreach presentation during team meetings to teach/inform GP nurses about the CDMIs (including an information sheet and test-account)	
Provide training to GP nurses in using the CDMIs and guiding patients	
• Two visits: a training of *2* h and a follow-up meeting of *1* h (approximately 2 months after the training visit). The goals of the visits are to target: ◦ Knowledge about the CDMIs, the recruitment and guiding process, skills to use the intervention, creating self-efficacy and a positive attitude, motivation, intentions to use the CDMIs ◦ Training topics: 1. Providing information about the CDMIs, the related monitoring system and guidelines for the recruitment and guidance process 2. Practising with the use of the CDMIs and monitoring system 3. Discussing possible barriers and facilitators for implementation • With respect to guidance, flexible guidelines were given to promote maximum adaptability to local needs and procedures of GP nurses. Guidelines include:Highlighting the benefits of the CDMIs with guidance, explaining the content and goal of the intervention, discuss the desirable frequency and content of guidance sessions (suggestions for topics are given in the manual), examples of motivational interviewing techniques, monitor and discuss progress in an interim evaluation, final evaluation of the intervention (process) • The follow-up meeting is aimed at: discussing first experiences, barriers and facilitators, repeating the main elements of the way recruitment and guidance is being given.	
Create and provide educational materials to GP nurses	
• Developing manuals, and other supporting materials (PowerPoint presentation, flowchart) in ways that make it easier for GP nurses to learn about the CDMIs and for GP nurses to learn how to deliver the CDMIs to patients. The educational materials contain information on: The usefulness of blended e-health, the content of the CDMIs, the monitoring system, steps and guidelines to be taken in the recruitment and guidance process (including motivational interviewing techniques), the study flowchart and patient information	
Facilitate exchange of information and experiences among GP nurses	
• Organising at least two implementation team meetings per participating organisation during the year following the training and follow-up visits (led by the research teams). The participating GP nurses will be given time to reflect upon and share their experiences, share lessons learned and support each other’s learning. Moreover, the research team can provide additional information, support or solve problems as needed. The aim is to contribute to increasing motivation, self-efficacy, and intentions to use the CDMIs and to remove potential barriers for implementation	
Facilitate a process of interactive problem solving and support for GP nurses	
• Providing a helpdesk (by telephone or email) for the GP nurses for either technical questions or questions about the implementation process, and actively providing information and tips to GP nurses through newsletters, telephone calls and emails. The aim is to contribute to increasing motivation, self-efficacy and intentions to use the CDMIs and to remove potential barriers for implementation	
Create awareness among patients about the CDMIs and the implementation project	
• Providing an information brochure and letter for patients to inform them about the online CDMIs and the research study	
Facilitate and encourage the use of the CDMIs among patients	
• Making adjustments to CDMIs to make them user friendly (e.g. providing reminders for use, simplifying some of the navigation and texts) • Providing additional information about the CDMIs and offering the opportunity to ask questions during the GP nurse consultation • The GP nurse can also show a demo video in which the content and goal of the online CDMIs are explained.	
Motivate and provide support to patients when using the online CDMIs	
• Providing guidance at each step during the intervention, if necessary. The aim is to teach the necessary skills to use the intervention, and create a positive attitude and motivate patients to use the CDMIs. • Guidance is provided according to the regular implementation strategy guidelines described above	

##### SES-sensitive implementation strategy

The SES-sensitive implementation strategy will encompass all the topics described in the regular implementation strategy, but will be tailored to match the needs of the lower-SES target group.

The GP nurses will receive a 3-h training (instead of a 2-h training), and follow-up meeting of 1.5 h (approximately 2 months after the training visit) in which extra elements of the SES-sensitive implementation strategy will be addressed. This additional section of the training was developed in collaboration with a representative of the Dutch Centre of Expertise on Health Disparities (Pharos). An important component is the recognition and guidance of lower-SES patients who might benefit from the online CDMIs. GP nurses will be taught about the concept and consequences of health-literacy skills and ways to recognise and align with the needs of patients with a lower SES or low/limited health-literacy skills. They will practice with methods to provide support to patients with lower SES or health-literacy skills (e.g. easier language and communication, teach-back method). They will also be strongly advised to provide a more directive approach to guiding patients and encouraging them to use the CDMIs. Practical tips will be given to do this, such as: not immediately offering the online CDMIs during the first visit, but after trust and alliance have been established, seeking contact more often (face-to-face, by telephone or monitoring system) to provide guidance, helping patients create an account, indicating which exercises the patient should do (an overview of exercises and suggestions is given in the manual), doing the online exercises with patients during face-to-face sessions before doing them alone (modelling), focussing on one or a few exercises to reduce the chance of experiencing failure, using appropriate language and checking whether information is understood (e.g. using the teach-back method).

The GP nurses in this condition will also have access to a helpdesk service, at least two implementation team meetings and ongoing communication activities from the research teams, but special attention will be paid to the lower-SES target group in all these actions.

Additional elements of the SES-sensitive implementation strategy aimed at the patients are:

- ▪ Extra guidance from the GP nurse, when needed, during the registration process (creating an account, username/password, intake questionnaire, etc.)
- ▪ Extra guidance from the GP nurse, if necessary, with the exercises (e.g. the GP nurse and the patient practice the exercise together during a consultation)
- ▪ A more proactive approach of delivering support by the GP nurse (e.g. extra reminders, extra contact moments by email or phone)

### Outcome measures

#### Primary outcome

The (difference in the) participation rate of patients with a lower SES in the CDMIs is the primary outcome. Participation is defined as participating in at least one face-to-face session with a GP nurse (as registered by the GP nurse) and engaging with at least two exercises in the online CDMIs (as determined with the user data of the online CDMIs). It is hypothesised that in the SES-sensitive implementation strategy arm of the trial the participation rate of lower-SES patients will be significantly higher than in the regular implementation strategy arm. This outcome requires an operational definition of SES, but SES can be defined in various ways. Indicators that are commonly used are education, income and work status/occupation [31–35]. Education is a widely used indicator, especially in the Netherlands, and has some advantages over the other two indicators [36–38]. First, the level of education is generally stable after early adulthood, whereas work status and level of income fluctuate more. Second, in general, respondents are willing to report their level of education, while respondents might be unwilling to disclose or even misstate the level of their income [38, 39]. For these reasons, education will be the main indicator of lower SES in this study. In addition to education level, income, work status and status level of the neighbourhood will be taken into account because these indicators cover different dimensions of SES and are, although interrelated, not interchangeable with education [33, 34].

Therefore, in this study, a lower SES will be defined as:

1. The highest completed educational level is intermediate vocational education (i.e. between lower and higher vocational education, in Dutch: MBO in Dutch: MBO) or lower, and/or
2. The respondent is unemployed (looking for work) and lives in a neighbourhood with a negative status level score, and/or:
3. The total gross family income of the respondent is below the social minimum income in the Netherlands (per July 2017), and lives in a neighbourhood with a negative status score

Self-reported education will be assessed with a single item (What is your highest completed educational level?) with eight response options: none/primary school, lower vocational education, intermediate secondary education, higher secondary education, intermediate vocational education, higher vocational education, academic education, and other type of education. We use the term ‘lower SES’ in this study as we include intermediate levels of education in our definition of lower SES. In the Netherlands, four levels of intermediate vocational education are discerned. Persons who report an intermediate vocational education as their highest completed education level will be asked to indicate which type of intermediate vocational education they completed (answer options correspond to the four levels). Generally, the first level is used as the cut-off for highest completed educational level in a low-SES definition (e.g. by Statistics Netherlands). However, we use the fourth level as the cut-off for highest completed educational level. We chose a higher cut-off point because it is mainly the highly educated (i.e. higher vocational education and academic education) that are currently reached with e-health interventions, despite the risk of depression generally increasing as educational level decreases [8].

Self-reported gross family income will be assessed with a single item. In the Netherlands, the social minimum income is dependent upon living arrangements (persons who live alone or as a single parent: rounded to an average of €1100 for this study, and for persons with any other type of living arrangement: €1550) which is reflected in the answer options: more or less than €1100 or more or less than €1550. Self-reported work status will be assessed with a single item using the following answer options: employed/self-employed, unemployed, occupationally disabled, student, volunteer work, retired, homemaker. Status level scores of the neighbourhood will be assessed by asking respondents their four-digit zip code. The Netherlands Institute for Social Research provides status scores for every zip code. For this study, the status level scores of 2016 will be used. A negative status level score indicates a lower than the Dutch average status level score of the years 1998–2016.

### Secondary outcomes

#### Psychological complaints

In line with the previous study on the effectiveness of the self-guided online CDMIs, we assessed the complaints targeted by the CDMIs and additional psychological outcomes as secondary outcomes.

- ▪ Depressive complaints will be measured using the 8-item Patient Health Questionnaire (PHQ-8) [40]. Items can be scored from 0 (not at all bothered) to 3 (bothered nearly every day) which results in a total score ranging from 0 to 24. Higher scores correspond to higher levels of depressive complaints. Sum scores of 5, 10, 15 and 20 represent cut-off points for mild, moderate, moderately severe and severe depression, respectively
- ▪ Sleep problems will be measured with the Jenkins Sleep Evaluation Questionnaire (JSEQ) [41]. It consists of four items that are scored on a 6-point scale (0 = not at all, 5 = 22–31 days) and can be added to obtain a total score ranging from 0 to 20. Higher scores indicate more sleep problems
- ▪ Stress will be measured with the 10-item Perceived Stress Scale (PSS-10) [42]. Items are scored on a 4-point scale (0 = never, 4 = very often), resulting in a total score ranging from 0 to 40. Higher score indicate higher levels of stress
- ▪ Worry will be assessed using the 11-item Penn State Worry Questionnaire (PSWQ) [43]. Items can be scored from 0 (not at all typical of me) to 5 (very typical of me) which results in a total score ranging from 11 to 55. Higher scores correspond to higher levels of worry
- ▪ Anxiety will be measured with the Generalised Anxiety Disorder Scale (GAD-7) [44]. The seven items are rated on a 4-point scale (0 = not at all sure, 4 = nearly every day). Items are summed which results in a total score (range 0–28), with higher scores defining a higher level of anxiety severity
- ▪ Well-being will assessed with the 5-item World Health Organisation Well-Being Index (WHO-5) [45]. The items can be scored from 0 (at no time) to 5 (all of the time) which results in a total score ranging from 0 to 25. Higher scores correspond to higher levels of well-being

#### Implementation-related factors

##### Health literacy

To increase the uptake of self-management interventions among people with a lower SES it may be important to take their health-literacy skills into account. Health-literacy skills, defined by the WHO as ‘the cognitive and social skills which determine the *motivation* and *ability* of individuals *to gain access to, understand and use information* in ways to promote and maintain good health’, play a crucial part in empowering people to use self-management interventions [23, 46]. Low or limited health-literacy skills are more prevalent among vulnerable groups, including people with a low education and a low income [22, 47, 48]. Examples of ways to pay attention to health-literacy skills in the implementation of interventions are: increasing the skills of health care providers to recognise low health-literacy skills, adapting communication into an easier understandable way, and helping people navigate through an intervention [49].

Health literacy will be assessed using the 14-item Dutch Functional Communicative and Critical Health Literacy Scale (FCCHL) [50, 51]. The items are scored on a 4-point scale (1 = never to 4 = always). Items can be summed and divided by the total number of items to obtain a total score ranging from 1 to 4, with higher scores indicating higher levels of health literacy.

##### Satisfaction and feasibility

Patients in both implementation groups will be asked about the usefulness and satisfaction with the intervention (e.g. ease of use, effectiveness in reducing/handling complaints, relevance of exercises, and other content, overall satisfaction) at the 3- and 12-month follow-ups. They will also be asked about the usefulness and satisfaction with the guidance they were provided with at the 3-month follow-up. In addition, in-depth interviews (*n* = 10) will be conducted to illicit patient experiences with the intervention, their preferences for the delivery of the online CDMIs in primary care and factors that they deem relevant for the sustained (successful) implementation of the intervention.

GP nurses will be asked about their experiences and satisfaction with the online intervention and guiding patients in using the intervention. They will be asked to rate the implementation materials (including the monitoring system and manual), their overall opinion of the intervention, which strategies they used to guide patients (with a high and lower SES), and how competent they felt in guiding patients with a lower-SES level. Based on the Unified Theory of Acceptance and Use of Technology (UTAUT model) [52]. GP nurses were asked to rate statements (1 = totally agree, 5 = totally disagree) relating to five dimensions that may directly or indirectly impact the use of technology according to the model: performance expectancy (four items), effort expectancy (four items), social influence (four items), facilitating conditions (four items) and behavioural intention to use the intervention (seven items). Moreover, statements relating to GP nurse attitudes (four items) and self-efficacy (two items) with respect to (their ability in) using the intervention will be included. Like patients, GP nurses will also be invited to participate in in-depth interviews (*n* = 10) to gain insight into their experiences with implementing the intervention in their daily practice, providing guidance, and facilitators and barriers for (further) implementation.

##### Fidelity and adherence

Adherence to the intervention by patients will be assessed with user data that are collected in the online CDMI system (i.e. the number of number of completed exercises and diary/monitor entries). The fidelity of the GP nurse to the intervention will be assessed by questioning GP nurses in the follow-up questionnaires about the guidance that they have given (e.g. the rate to which they recommended exercises, the type of exercises recommended, the intensity of the guidance). Furthermore, GP nurses will be asked to report per patient about the number of face-to-face consultations. These data will also be used to provide some insight into the costs of the intervention. For costing we shall make use of the pertinent Dutch guideline for economic evaluation and rely on the standard cost prices reported therein. These will be indexed to the study’s reference year 2017. The user data of the monitoring system will be used to evaluate the use of this tool by GP nurses.

#### Additional factors

##### Sociodemographic factors

In addition to income, work status and neighbourhood status (see above), other sociodemographic factors that will be assessed are: age, gender (male/female), living arrangement (alone, with partner, with partner and children, without partner and with children, with parents, other), marital status (single, married, civil partnership, divorced, widowed) and country of birth.

Data will be assessed in all patients, not only lower-SES patients. First, secondary outcomes (such as impact on complaints and well-being) will primarily be examined for within-group differences, but we are also interested in between-group differences. Second, because ‘low SES’ and ‘participation’ can be defined in various ways we will conduct additional analyses for which data from all patients are needed. Lastly, in order to compare outcomes with other studies, secondary outcomes of all patients must be assessed.

### Sample size calculation

The sample size is based on the main outcome, namely participation rate of patients with a lower SES. This is defined as participating in at least one face-to-face session with a GP nurse and completing two exercises in the online CDMIs (see above). We expect a (base) participation rate of 18% with the regular implementation strategy. Due to a paucity of studies about online psychological interventions offered and guided by primary care professionals, this percentage is based on previous (baseline) participation rates of lower-educated people in studies of online self-help interventions for depression [18, 20]. This percentage also takes into account that our definition of participation entails more than just starting with the intervention/trial and that attrition in e-health can be related to indicators of lower SES [9]. We aim to double the participation rate of lower-SES patients to 36% by using the SES-sensitive implementation strategy; a smaller effect was not deemed to be relevant. As far as we know, there is no empirical evidence that might support this doubling; however, our rationale is that with specific training and specific materials this percentage of participating lower-SES patients can be reached. The difference between these fractions (0.18 vs. 0.36) will be tested at a two-sided significance level of 5% and a power of 80%. Accounting for the clustering in the data with six patients on average per GP nurse (range 2–14 patients) and an intraclass correlation of 0.02, we need *n* = 114 patients in each condition (in 19 GP nurses per condition) or 228 participants in total. This normative sample size calculation was performed in Stata version 14.2 statistical software package using the clustersampsi-procedure, taking into account the mean cluster size, cluster size variation and the intraclass correlation [53]. The intraclass correlation value of 0.02 was based on the Hospital Anxiety and Depression Scale (HADS)-depression scores in the primary care setting as reported by Adams and colleagues (2004), Table 5 [54].

### Statistical analysis

#### Primary outcome

The effect of the implementation strategies on lower-SES participation will be analysed using mixed-effects logistic regression models. This analysis technique allows for the clustering of patients within primary care practices, for dependence of observations within individuals over time, and provides intention-to-treat analysis as required by the Consolidated Standards of Reporting Trials (CONSORT) Statement. The allocated implementation strategy will be used as the fixed between-groups factor.

#### Secondary outcomes

##### Impact on complaints and well-being

Linear mixed models will also be used to analyse the impact of the implementation strategies on the (secondary) psychological complaint outcomes (i.e. depression, sleep, stress, worry and anxiety) and well-being. In this instance our interest is not in comparing between-group differences on complaint-related outcomes, as both groups will receive the online CDMI intervention (albeit with a different type of implementation strategy). However, we are interested in examining whether there are within-group differences in clinical outcomes (i.e. reductions in complaints) between baseline and follow-up, especially in the SES-sensitive implementation group. For this purpose, growth curve analysis will be performed to examine the within-group trajectories of the complaint-related outcomes. Again, clustering will be accounted for in these analyses.

##### Health literacy

To examine bivariate associations between health literacy and the participation rate and between FCCHL and process measures, independent *t* tests and chi-square statistics will be used.

##### Costs

In the context of this implementation study, the cost analysis will have to be very simple and descriptive in nature. Our cost analysis will be restricted to just mapping (and quantifying) the extra financial costs for rolling out the SES-sensitive implementation strategy as compared to opting for the regular implementation strategy.

##### Process indicators of implementation

Descriptive statistics will be obtained on satisfaction, feasibility, fidelity and adherence with the intervention. In line with the UTAUT model, relationships between performance expectancy, effort expectancy, social influence, facilitating conditions, and behavioural intention to use the intervention will be explored in regression models. Interviews with GP nurses and patients will be transcribed verbatim. The transcripts will be analysed by coding the texts using qualitative analysis software (MaxQDA) and themes concerning potential improvements to the blended online CDMIs and impeding and facilitating factors for (sustained) implementation will be identified and described.

#### Additional analyses

Demographic factors will be used to describe the characteristics of the study sample at baseline. Attrition analysis will be conducted by comparing demographic characteristics of the patients who participated in the blended online CDMIs vs. the non-participants. Also, demographic characteristics and psychological complaint outcome variables of the participants who completed the follow-up questionnaires with those who did not complete these assessments will also be compared. For the purpose of attrition analysis, differences between the groups will be tested using independent *t* tests and chi-square statistics.

Because (lower) SES can be defined in various ways and there is no clear common definition, we will conduct additional sensitivity analyses with (lower) SES as outcome defined in other ways. For example, we will use a lower cut-off for educational level (MBO4 and/or MBO1 instead of MBO-4) to define lower SES. We will also examine the use of more than two exercises as an alternative in the definition of participation.

Exploratory analyses, with an interaction term, will be conducted to examine whether health literacy modifies the effect of implementation strategy on participation of lower SES patients. We will also explore the user data of the participants to see if lower SES patients (in both group) use the CDMIs differently than higher-SES patients.

## Discussion

A report on prevention in Dutch health care [55] suggested that integrating preventive e-health within primary care would be a necessary and important precondition to increase the reach of depression prevention. It explicitly suggests that the GP and the GP nurse should expand their therapeutic arsenal by offering blended care, i.e. expand their face-to-face care with Internet modules. Exactly how this should be done and which implementation strategies would be best is currently unclear. This is particularly relevant for lower SES groups because of their lower participation rate in e-health interventions, despite their elevated risk developing mental health disorders [8]. There is a clear need to develop a dedicated implementation strategy to implement effective e-health for depressive complaints, in order to increase their reach, especially among disadvantaged and lower-SES groups. It is, therefore, also important to empirically test whether a dedicated SES-sensitive implementation strategy will actually increase the participation rate of this group of patients. A strength of our study is that a regular and a dedicated implementation strategy will be compared in a randomised implementation trial. As noted before, defining SES in general and lower SES in particular is complex. We will, therefore, use a composite measure to define lower SES which takes various factors (i.e. education, work status, income, and the SES of the neighbourhood) into account to tackle this issue. Moreover, in this study we focus on lower SES (i.e. including medium education levels) as opposed to only low SES due to the fact that, at the moment, especially highly educated individuals seem to use e-health. For this reason, our primary focus is on improving participation rates in a broader group of lower SES patients. However, we have planned sensitivity analyses to examine different operational definitions of low(er) SES, including using a lower cut-off for education level than in the primary analysis, in order to determine the impact of the implementation strategies when other definitions of low SES are used.

In conclusion, this study should contribute to our knowledge of reaching lower SES groups with an evidence-based brief and complaint-specific blended approach for depressive complaints in primary care. It should also further our knowledge on successful strategies to implement online depression prevention in primary care in general.

## Trial status

Patient recruitment started in January 2018 and is expected to be completed in June 2019. This study was approved by the VU Medical Center Ethics Committee (reference number 2017.437). The protocol under approval is version 1.0 (22 August 2017).

## Supplementary information

**Additional file 1.** SPIRIT 2013 Checklist: recommended items to address in a clinical trial protocol and related documents.

## Data Availability

Data sharing is not applicable to this article as no datasets were generated or analysed during the current study.

## Abbreviations

CDMI: Complaint-Directed Mini-Interventions
FCCHL: Functional Communicative and Critical Health Literacy Scale
GAD-7: 7-item Generalised Anxiety Disorder Scale
GP nurse: General practice nurse
GP: General practitioner
HADS: Hospital Anxiety and Depression Scale
JSEQ: Jenkins Sleep Evaluation Questionnaire
MBO: Intermediate vocational education (Dutch: Middelbaar Beroepsonderwijs)
PHQ-8: 8-item Patient Health Questionnaire
PHQ-9: 9-item Patient Health Questionnaire
PSS-10: 10-item Perceived Stress Scale
PSWQ: Penn State Worry Questionnaire
SES: Socioeconomic status
UTAUT: Unified Theory of Acceptance and Use of Technology
WHO-5: 5-item World Health Organization Well-Being Index
WSQ: Web Screening Questionnaire
